# Exome sequencing identifies *NFS1* deficiency in a novel Fe-S cluster disease, infantile mitochondrial complex II/III deficiency

**DOI:** 10.1002/mgg3.46

**Published:** 2013-11-18

**Authors:** Sali M K Farhan, Jian Wang, John F Robinson, Piya Lahiry, Victoria M Siu, Chitra Prasad, Jonathan B Kronick, David A Ramsay, C Anthony Rupar, Robert A Hegele

**Affiliations:** 1 Robarts Research Institute, Schulich School of Medicine and Dentistry, Western UniversityLondon, Ontario, N6A 5K8, Canada; 2 Department of BiochemistrySchulich School of Medicine and Dentistry, Western UniversityLondon, Ontario, N6A 5C1, Canada; 3 Medical Genetics ProgramDepartment of Pediatrics, London Health Sciences CentreLondon, Ontario, N6C 2V5, Canada; 4 Children's Health Research Institute, London Health Sciences CentreLondon, Ontario, N6C 2V5, Canada; 5 Division of Clinical and Metabolic GeneticsThe Hospital for Sick ChildrenDepartment of Pediatrics, University of TorontoToronto, Ontario, M5G 1X8, Canada; 6 Department of Pathology, London Health Sciences CentreLondon, Ontario, N6A 5A5, Canada

**Keywords:** Autozygosity mapping, Fe-S proteins, mitochondrial complex deficiency, *NFS1*, whole-exome sequencing.

## Abstract

Iron-sulfur (Fe-S) clusters are a class of highly conserved and ubiquitous prosthetic groups with unique chemical properties that allow the proteins that contain them, Fe-S proteins, to assist in various key biochemical pathways. Mutations in Fe-S proteins often disrupt Fe-S cluster assembly leading to a spectrum of severe disorders such as Friedreich's ataxia or iron-sulfur cluster assembly enzyme (ISCU) myopathy. Herein, we describe infantile mitochondrial complex II/III deficiency, a novel autosomal recessive mitochondrial disease characterized by lactic acidemia, hypotonia, respiratory chain complex II and III deficiency, multisystem organ failure and abnormal mitochondria. Through autozygosity mapping, exome sequencing, in silico analyses, population studies and functional tests, we identified c.215G>A, p.Arg72Gln in *NFS1* as the likely causative mutation. We describe the first disease in man likely caused by deficiency in NFS1, a cysteine desulfurase that is implicated in respiratory chain function and iron maintenance by initiating Fe-S cluster biosynthesis. Our results further demonstrate the importance of sufficient NFS1 expression in human physiology.

Iron-sulfur (Fe-S) clusters are a class of ubiquitous prosthetic groups thought to be highly conserved in essentially all organisms (Sheftell et al. 2010). Their unique chemical properties allow the proteins that contain them to assist in vital and diverse biochemical pathways such as iron metabolism and homeostasis as well as DNA damage repair (Kispal et al. 1999; Rudolf et al. 2006; Lill et al. 2012). Eukaryotic Fe-S proteins are localized to the mitochondria (Lill et al. 2012), cytosol (Tong and Rouault 2006), endoplasmic reticulum (Lill et al. 2006), and nucleus (Naamati et al. 2009). Fe-S proteins initiate biogenesis of the Fe-S cluster complex, which can transfer electrons, act as catalysts, stabilize protein structures, and execute regulatory functions (Kispal et al. 1999; Rudolf et al. 2006). Given the central role of Fe-S clusters in many fundamental biochemical pathways, deficiency in genes encoding Fe-S proteins are expected to cause human disease. Classic examples of Fe-S cluster deficiency related to human disease include the following: Friedreich's ataxia (FRDA [MIM 229300]), the most common autosomal recessive ataxia due to a homozygous expansion of a GAA trinucleotide repeat in intron 1 of *FXN* (GenBank accession number, NM_000144.4) (Koeppen 2011) and ISCU myopathy [MIM 255125], due to a splicing defect in *ISCU* (GenBank accession number, NM_213595.2) that alters the C-terminus ultimately reducing protein expression (Kollberg et al. 2009). Other Fe-S cluster-related diseases include those caused by mutations in *NFU1* (GenBank accession number, NM_015700.3) or *BOLA3* (GenBank accession number, NM_212552.2), which ultimately disrupt Fe-S enzyme lipoate synthase activity (Cameron et al. 2011). This leads to a severe infantile multisystem disorder known as multiple mitochondrial dysfunctions syndrome, which is characterized by hyperglycinemia, lactic acidosis, hypertension, and failure to thrive (Cameron et al. 2011).

The interaction network of proteins involved in Fe-S cluster assembly is becoming clearer in both yeast and mammalian cells. In particular, NFS1 (MIM# 603485, GenBank accession number, NM_021100.4), ISCU, ISD11 (GenBank accession number, NM_020408.4), and FXN form the Fe-S cluster core complex (Prischi et al. 2010; Bridwell-Rabb et al. 2011; Schmucker et al. 2011; Rouault 2012). Fe-S cluster formation begins with NFS1 forming a homodimer to which monomers of a scaffold protein ISCU, bind near the top and the bottom (Shi et al. 2010). A cofactor pyridoxal 5' phosphate, helps NFS1 provide inorganic sulfur from cysteine residues, which then bind to cysteine ligands supplied by ISCU, that further covalently bind to iron (Raulfs et al. 2008). The core complex then recruits NFS1-binding protein, ISD11 (Adam et al. 2006) and finally FXN (Prischi et al. 2010). Next, the Fe-S cluster is transferred to recipient apo-proteins via binding of ISCU to chaperone proteins (Craig and Marszalek 2002). In short, it is clear that Fe-S cluster assembly is a highly conserved multistep process requiring cysteine desulfurases, scaffold proteins, chaperones, and iron donors to ultimately maintain iron homeostasis, execute catalysis, and regulate gene expression (Lill et al. 2012; Rouault 2012).

Here, we report a rare genetic mitochondrial disorder designated as infantile mitochondrial complex II/III deficiency (IMC23D), characterized by lactic acidemia, hypotonia, respiratory chain complex II and III deficiency, multisystem organ failure, and abnormal mitochondria affecting three children of healthy third cousins within an Old Order Mennonite community. The first child was admitted at 7 months of age. She was dehydrated and hypoglycemic after suffering a brief episode of pharyngitis. One day prior to admission, she presented with lethargy, anorexia, and oliguria. She had a generalized seizure followed by metabolic acidosis, respiratory decompensation, and severe myocardial failure. Her blood lactate, liver enzymes, and amylase levels were elevated (Table 1). She developed persistent hypotension, worsening oliguria, and peripheral edema. She died of cardiac failure 3 days post admission.

**Table 1:** Clinical and biochemical description of patients with IMC23D.

	Affected Individuals (year of birth)		
Clinical features	IV-I (1992)	IV-II (1994)	IV-III (1999)
Gestation	Full term	Full term	Full term
Gender	Female	Male	Male
Karyotype	46,XX	46,XY	46,XY
Facial/limb dysmorphology	−	−	−
Lethargy/anorexia/hypotonia	+	+	+
Autopsy	+	+	+
Age at last assessment	7 months, deceased	7 months, deceased	12 years
Height (cm)			154.9 (50th percentile)
Weight (kg)			50 (75th–90th percentile)
Associated diagnoses			
Respiratory failure	+	+	−
Cardiac failure	+	+	−
Hemorrhagic pancreatitis	+	−	−
Cerebral infarction	−	+*	−
Renal failure	+	+	−
Disseminated intravascular coagulation	+	+	
Seizures	+	+*	
Differential diagnoses			
Mitochondrial encephalomyopathy, lactic acidosis, and stroke-like episodes (MELAS), c.3243A>G	−	−	−
Myopathy, c.3260A>G	−	−	−
Cardiomyopathy, c.3303C>T	−	−	−
Myoclonic Epilepsy with Ragged Red Fibers (MERF), c.8344A>G	−	−	−
Neurogenic muscle weakness, Ataxia, and Retinitis Pigmentosa (NARP)/Leigh syndrome, c.9883T>G/C	−	−	−
Biochemical Findings			
Hypoglycemia	+	+	−
Increased lactate (normal: 0.5–2.2 mmol/L)	+	+	+ (ranged between 4 and 6 mmol/L)
Increased aspartate aminotransferase	+	+	+
Increased amylase	+	−	
Increased creatine kinase	+	+	+
Increased plasma amino acid concentration	+ (most amino acids)	+	+ (small increase in Alanine)
Urine organic acids	+	N	+
Amino aciduria	−	+	+
Metabolic acidosis	+	+	+
Respiratory chain enzymes			
Muscle mitochondria (nmoles/min/mg)			
Complex I + III (range: 37–99, mean: 71)	23^a^	33^b^, 29^a^	
Complex II + III (range: 85–214, mean: 152)	5^a^	27^a^, 23^b^	
Complex IV (range: 193–354, mean: 264)	156^a^	181^b^, 111^a^	
Citrate synthase (range: 170–481, mean: 339)	229^a^	846^b^, 540^a^	
Liver mitochondria (nmoles/min/mg)			
Complex I + III (range: 2–14, mean: 7)	7^a^	19^b^	
Complex II + III (range: 18–70, mean: 45)	133^a^	13^b^	
Complex IV (range: 15–100, mean: 41)	39^a^	35^b^	
Citrate synthase (range: 15–53, mean: 33)	37^a^	14^b^	

N, normal; +, present; −, absent;

a autopsy;

b biopsy;

* at 15 weeks of age. Blank cells indicate unavailable information.

The second child was admitted with respiratory failure and cyanosis at 6 weeks of age. He was normoglycemic but had elevated levels of lactate, aspartate aminotransferase (AST), and creatine kinase (CK) (Table 1). At 15 weeks, he was readmitted with truncal hypotonia, feeding problems, vomiting, and acidosis. He developed multisystem organ failure including cardiorespiratory failure, which required intubation, ventilation, and inotropic support. He had focal seizures with cerebral infarction documented by computed topography, renal failure, and disseminated intravascular coagulation. He was treated with a mitochondrial cofactor therapy consisting of riboflavin (50 mg twice daily [BID]), coenzyme Q_10_ (30 mg BID), thiamine (50 mg BID), vitamin C (100 mg BID), vitamin E (400 IU once daily [OD]), and vitamin K (2 mg OD), which led to a rapid and substantial clinical and biochemical improvement allowing extubation and discharge from the hospital. He did continue to have mild hypotonia. At 7 months, symptoms reoccurred; he worsened rapidly and died of cardiac failure 3 days post admission, despite intensive treatment.

A third child was born to the couple and was monitored for the same suspected mitochondrial disease. Neonatal neurological examination revealed symmetrical mild hypotonia of the upper limbs and to a lesser extent, of the lower limbs. He had symptoms in infancy consistent with those observed in his siblings, which included hypotonia, lethargy, weight loss, and abnormal lactate levels (Table 1). Similarly, he was treated with a mitochondrial cofactor therapy from 6 months until about 11 years of age. He is generally healthy today. However, his blood lactate, AST, and CK levels remain elevated (Table 1). While he has no history of seizures or problems related to his heart, he still has some mild developmental and gross motor delay, consisting primarily of a minor tremor and overall low energy. The lactic acidemia has persisted albeit fluctuated, for ∼8 years but has recently abated. The results of metabolite analyses in all three children support the diagnosis of a mitochondrial disease.

No specific findings relevant to the molecular abnormalities were noted from the general autopsy materials. However, both cases (IV-I and IV-II) exhibited hepatic steatosis, a common nonspecific finding in various, including metabolic, disorders. Hemorrhagic pancreatitis with secondary mesenteric fat necrosis, a nonspecific complication of “critical illness,” was present in individual IV-I and there was evidence of acute myocardial ischemic injury in individual IV-II, a common agonal finding. No other definite histological abnormalities were noted in the myocardium although this does not exclude the presence of a significant mitochondrial disorder in the heart.

Under the light microscope, the majority of the muscle fibers in the biopsy from individual IV-II were unusually small and many of them contained small, faintly basophilic granules in the section stained with hematoxylin, phloxin and saffron; these granules were magenta in the section stained with gomori modified trichrome and exhibited increased enzyme activity in the sections treated with reduced nicotinamide adenine dinucleotide-tetrazolium reductase and cytochrome C oxidase histochemistry. The succinic dehydrogenase activity was extremely weak in comparison to normal controls. Focally prominent subsarcolemmal collections of periodic acid-Schiff-positive glycogen were observed (Fig. 1A).

**Figure 1:**
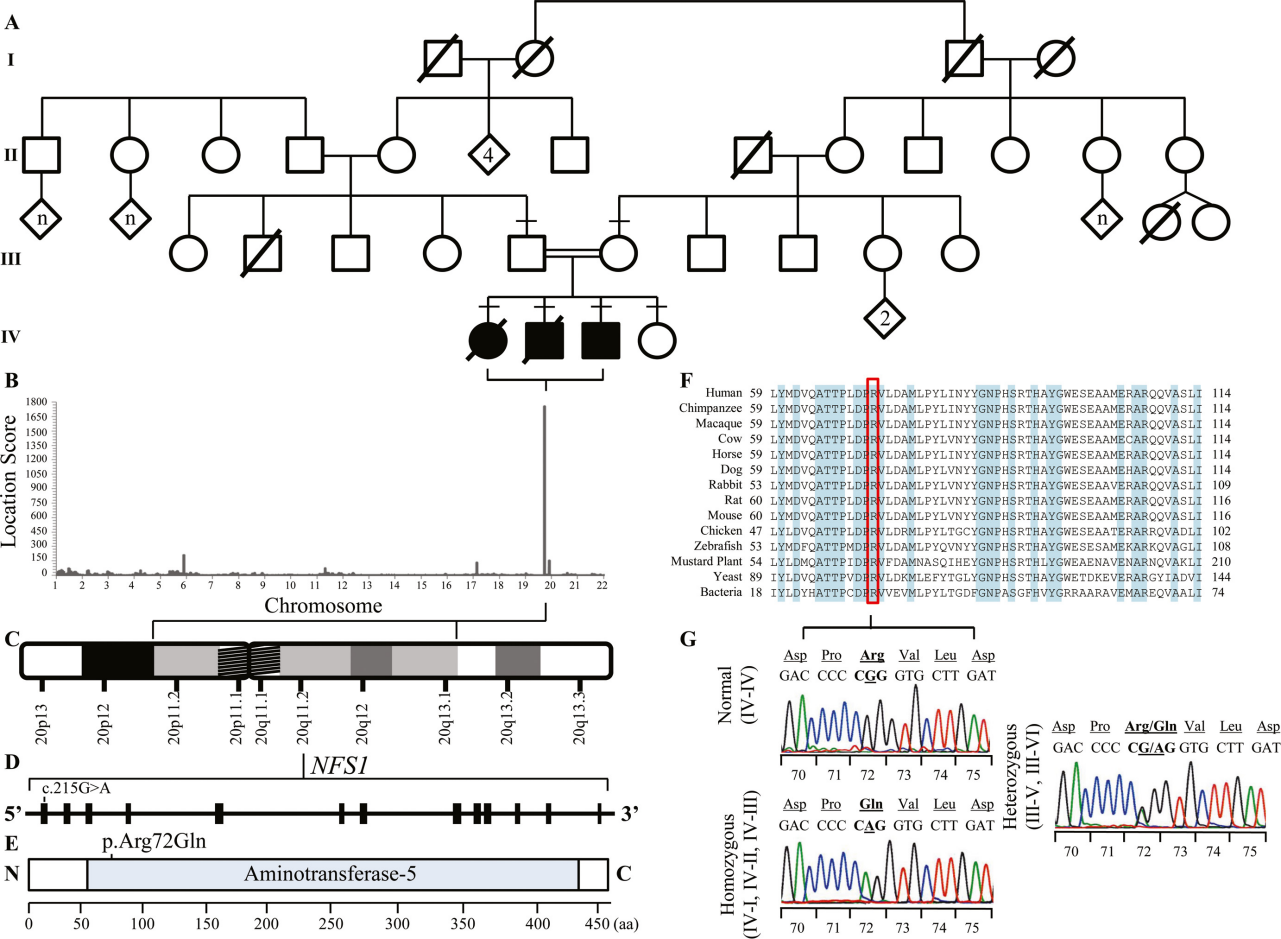
Mapping and exome sequencing of autosomal recessive infantile mitochondrial complex II/III deficiency (IMC23D) family identifies a highly conserved and destabilizing missense mutation, p.(Arg72Gln) in *NFS1*. (A) An Old Order Mennonite pedigree showing a union between two healthy third cousins. Three out of four children are affected with IMC23D. Affected individuals are shown in shaded squares (male) and circles (female). Diagonal lines across symbols indicate deceased individuals. A consanguineous marriage is shown by a double line between two individuals. Horizontal dashes above symbols indicate individuals who underwent DNA analysis. Diamonds indicate unspecified genders. (B) Genome-wide autozygosity mapping confirmed the autosomal recessive mode of inheritance by generating a long homozygous segment unique to the affected individuals on chromosome 20p11.2-q13.1 with a highly significant location score of 1754. (C) Ideogram depicting the homozygous segment unique to the affected individuals, Chromosome 20p11.2-q13.1, spanning 27.7 Mb. (D) The *NFS1* gene consists of 13 coding exons with a nonsynonymous nucleotide change, c.215T>G in exon one. Nucleotide numbering reflects cDNA numbering with +1 corresponding to the A of the ATG translation initiation codon in the reference sequence, according to journal guidelines (http://www.hgvs.org/mutnomen). The initiation codon is codon 1. (E) The structure of the NFS1 protein contains one domain, aminotransferase class V domain, shown from the N-terminal to C-terminal end. The amino acid (aa) p.Arg72 is harbored within the aminotransferase class V domain. (F) Multiple alignments demonstrate high conservation of the aa residue p.Arg72 across a set of species-specific NFS1 homologs. A ClustalW analysis of the NFS1 region encompassing the mutation site at residue p.Arg72 (highlighted in red) in aligned homologs with multiple divergent species is shown. The residues shaded in blue indicate fully conserved residues. (G) DNA sequence analysis of p.Arg72Gln from genomic DNA of a normal individual (IV-IV, top left electropherogram), a homozygous individual (IV-I, IV-II, IV-III, bottom left electropherogram), and a p.Arg72Gln heterozygous individual (III-V, III-VI, right electropherogram). For each electropherogram, amino acid codes are shown in the top line with nucleotide sequence and codon numbers below. The position of the mutated nucleotide is underlined.

Given the known consanguinity of the parents, we modeled an autosomal recessive mode of inheritance. Blood and tissue samples were collected from the family following appropriate and informed consent in accordance with the Research Ethics Board at Western University. The detailed materials and methods can be found elsewhere (see Methods in Data S1). Genome-wide autozygosity mapping generated a locus on chromosome 20p11.2-q13.1, ∼27.7 Mb with a highly significant location score of 1754 (Fig. 2B). The long segment of homozygosity was unique to the affected individuals and contained 4239 single nucleotide polymorphisms (SNPs) belonging to 453 genes bordered by SNPs rs4305333:A>C and rs6125184:A>G, which correspond to 20:18,891,229 and 20:46,576,714, respectively (Fig. 2–A–C). Next, we applied whole-exome sequencing and identified a novel variant within the autozygous region: c.215G>A, p.Arg72Gln in *NFS1*, a highly conserved cysteine desulfurase involved in the Fe-S cluster assembly machinery and is essential for the maturation of other Fe-S proteins. Nucleotide numbering reflects cDNA numbering with +1 corresponding to the A of the ATG translation initiation codon in the reference sequence, according to journal guidelines (http://www.hgvs.org/mutnomen). NFS1 p.Arg72Gln cosegregates with disease status in the family and was consistently predicted to be damaging by multiple in silico analyses. Exome sequencing identified additional coding variants within other genes in the autozygous region with a minor allelic frequency (MAF) >1%, however, given the rarity of the disease, these variants were not further analyzed (Table S1).

**Figure 2:**
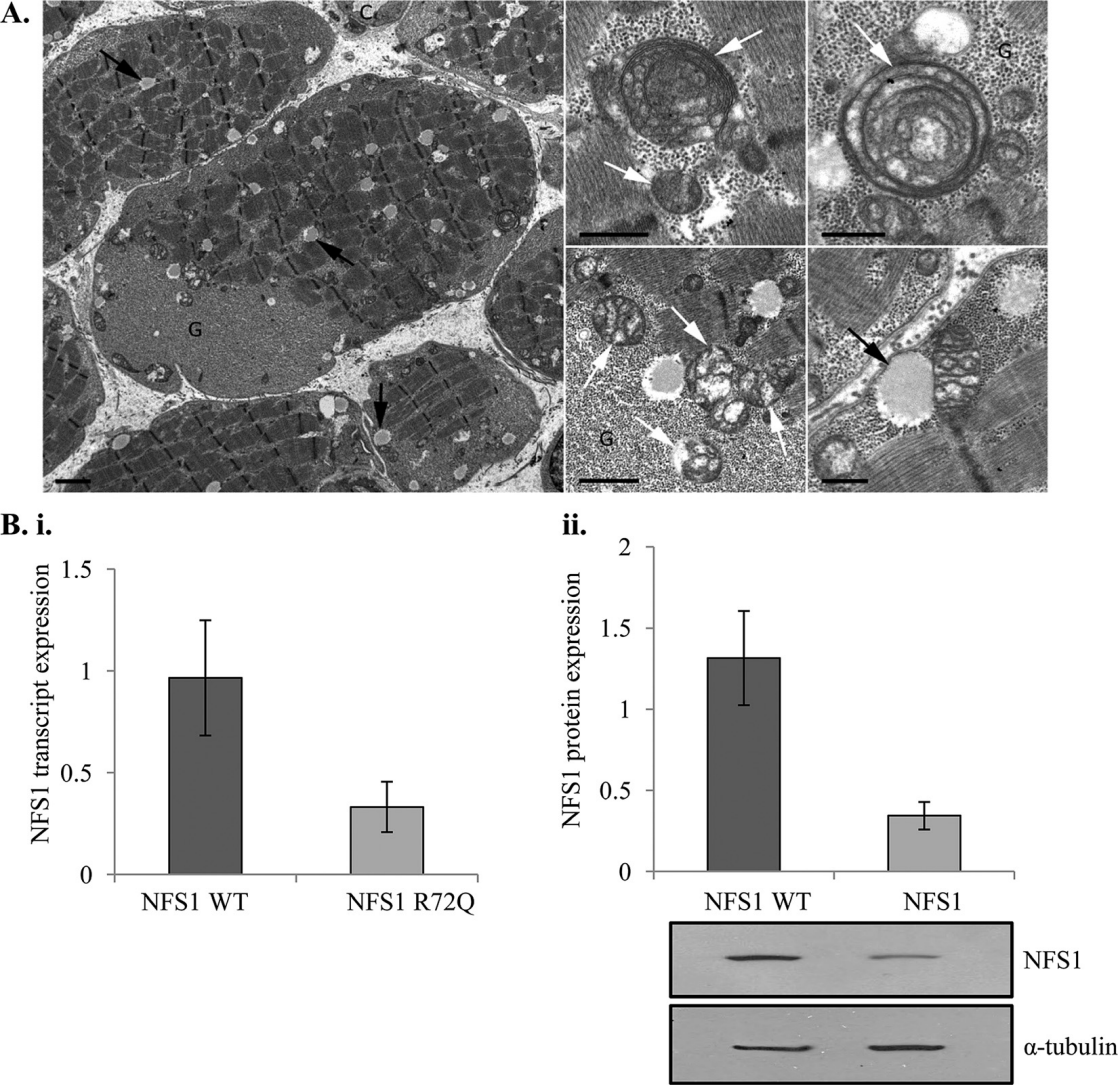
Pathological and cellular dysfunction in patients with infantile mitochondrial complex II/III deficiency (IMC23D). (A) Pathological findings in patients with IMC23D. These electron micrographs show scattered lipid droplets (black arrows) and abundant glycogen (G), which are common nonspecific findings. Note also the lack of capillaries (left) – there should be at least three but only one can be seen (C). The lipid droplets are often close to directly or attached to the mitochondria (bottom right). There are a variety of mitochondrial abnormalities (white arrows), including concentric cristae (top middle and right), a honeycomb arrangement of the cristae (top middle and right, bottom middle), vacuolated or ‘blown’ mitochondria reminiscent of artifacts but possibly also evidence of ‘metabolic fragility’ (bottom center), and finally scant cristae (bottom right). Scale bars 2* μ*m (left), 1 *μ*m (bottom center), 500 nm (top center and right, bottom right). (B) Depletion in NFS1 protein and transcript levels in patients with IMC23D. Both (i) quantitative PCR and (ii) Western analysis show reduced NFS1 cellular expression in fibroblast cells of patients affected with IMC23D. Bar graphs indicate means ± standard deviations from two sets of experiments, showing the relative NFS1 expression in affected individuals normalized to a healthy control quantified by densitometry. The autoradiographs provide a visual representation of NFS1 protein level. The upper blot shows decreased protein expression in NFS1 R72Q relative to NFS1 wild-type (WT). The lower blot shows the constitutive expression of *α*-tubulin in both experiments.

To determine the MAF, we began by genotyping 40 otherwise healthy controls from the Old Order Mennonite community. We did not identify any carriers for *NFS1* c.215G>A, p.Arg72Gln, however, after genotyping 3033 additional individuals from an ethnically diverse cohort, we identified one heterozygote (0.016%), which was consistent with the reported MAF in the NHLBI ESP Exome Variant Server. Furthermore, we performed quantitative reverse transcriptase-polymerase chain reaction (qRT-PCR) and Western analysis using fibroblast cells from the affected individuals and observed markedly reduced NFS1 transcript and protein levels relative to those from a healthy control (Fig. 3B). Using enzymology assays on muscle and liver tissue, we saw deficiency in mitochondrial respiratory chain complex II and III, which is consistent with decreased NFS1 protein levels and likely, a disruption in Fe-S cluster assembly (Table 1). Lastly, when wild-type and patient fibroblast cells were subjected to co-immunoprecipitation with an antibody against NFS1, ISD11 was co-precipitated with NFS1 in wild-type but not in patient cells (Fig. S1). This suggests that the NFS1-ISD11 complex is disrupted in patient cells. Interestingly, mutations in *LYRM4*, which encodes ISD11, also result in a compromised NFS1-ISD11 complex and leads to a clinical presentation similar to IMC23D (Lim et al. 2013).

During our analysis, we identified a second variant within the autozygous region, c.499C>T, p.Arg167Cys in *CDH22* (GenBank accession number, NM_021248.2), a calcium-dependent cell adhesion protein predominately expressed in the brain (Sugimoto et al. 1996; Saarimaki-Vire et al. 2011). *CDH22* p.Arg167Cys was predicted to be pathogenic by in silico analyses, cosegregated with disease status in the family and has a MAF of 0.062% according to NHLBI ESP Exome Variant Server. We performed qRT-PCR and Western analysis to determine the effect of p.Arg167Cys in *CDH22* on protein function, however, we were unable to measure any CDH22 transcript and protein levels in patient fibroblast cells. Given the nature of the protein in that it is predominantly expressed in the brain, and has not been linked to mitochondrial dysfunction, we believe p.Arg167Cys in *CDH22* is not involved in IMC23D and is in linkage disequilibrium with *NFS1* p.Arg72Gln.

Until now three Fe-S proteins, namely NFS1, NBP35 (GenBank accession number, NM_002484.3), and IOP1 (GenBank accession number, NM_022493.1), had not been linked to a human deficiency state (Sheftell et al. 2010). There are arguably two reasons for this: (1) such disorders have not yet been ascertained; or (2) a deficiency in these vital and well-conserved gene products is incompatible with life. Several studies have examined the cellular localization of NFS1, its role in Fe-S cluster biogenesis and assembly, as well as its role in other pathways such as donating sulfur to MOCS3 in the molybdenum cofactor biosynthesis pathway (Marelja et al. 2008). However, two studies examined the physiological consequence of cellular NFS1 depletion via small interfering RNA (siRNA)-mediated gene silencing approaches and are of particular interest (Biederbick et al. 2006; Fosset et al. 2006). In one study, depletion of mouse NFS1 (m-Nfs1) in murine fibroblasts led to reduced activity of mitochondrial respiratory chain complex I and II and citric acid cycle protein, aconitase (Fosset et al. 2006). Interestingly, there was no change in mitochondrial malate dehydrogenase activity or cytosolic lactate dehydrogenase, neither of which contain Fe-S clusters (Fosset et al. 2006). The second study showed severe growth retardation and morphological changes in mitochondria following NFS1 gene silencing in HeLa cells (Biederbick et al. 2006). Similarly, it showed decreased activity in both mitochondrial and cytosolic Fe-S proteins (Biederbick et al. 2006). Interestingly, introduction of m-Nfs1 repaired growth and restored Fe-S protein activity (Biederbick et al. 2006). These findings are consistent with an NFS1-dependent human deficiency state, as seen in our patients. Studies of this type together with our findings shed light on the physiological significance of proper Fe-S cluster biogenesis and assembly and their role in human health particularly, Fe-S cluster-related diseases.

## Supporting Information

Figure S1 NFS1-ISD11 interaction is disrupted in patient cells. Wild-type and patient fibroblast cells were subjected to co-immunoprecipitation with an antibody against NFS1. ISD11 was co-precipitated with NFS1 in wild-type (NFS1 WT) but not patient cells (NFS1 R72Q).

Table S1 Additional rare coding variants (MAF < 5%) identified within the autozygous region, chromosome 20p11.2-q13.1.
