# Supplementary material for: Exome sequencing identifies NFS1 deficiency in a novel Fe-S cluster disease, infantile mitochondrial complex II/III deficiency
Source: Mol Genet Genomic Med. 2013 Nov 18;2(1):73–80. doi: 10.1002/mgg3.46 (PMC3907916; doi:10.1002/mgg3.46)
Supplement: Figure S1 — NFS1-ISD11 interaction is disrupted in patient cells. Wild-type and patient fibroblast cells were subjected to co-immunoprecipitation with an antibody against NFS1. ISD11 was co-precipitated with NFS1 in wild-type (NFS1 WT) but not patient cells (NFS1 R72Q). [file mgg30002-0073-sd1.docx]

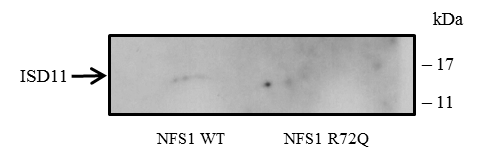


**Supplementary Figure 1.** NFS1-ISD11 interaction is disrupted in patient cells. Wild-type and patient fibroblast cells were subjected to co-immunoprecipitation with an antibody against NFS1. ISD11 was co-precipitated with NFS1 in wild-type (NFS1 WT) but not patient cells (NFS1 R72Q).
