# Supplementary material for: Exome sequencing identifies NFS1 deficiency in a novel Fe-S cluster disease, infantile mitochondrial complex II/III deficiency
Source: Mol Genet Genomic Med. 2013 Nov 18;2(1):73–80. doi: 10.1002/mgg3.46 (PMC3907916; doi:10.1002/mgg3.46)
Supplement: Table S1 — Additional rare coding variants (MAF < 5%) identified within the autozygous region, chromosome 20p11.2-q13.1. [file mgg30002-0073-sd2.docx]

**Supplementary Table S1. Additional rare coding variants (MAF <5%) identified within the autozygous region, chromosome 20p11.2-q13.1.**

| **Gene** | **cDNA change** | **Amino acid change** | **Type of mutation** | **MAF (%)** | ***in silico* prediction** | **Gene function** |
| --- | --- | --- | --- | --- | --- | --- |
| *RPN2*  (GenBank accession number, NM_002951.3) | c.1025G>A | p.Gly374Asp | Missense | 2.30 | Likely benign | Post-translational modification |
| *C20ORF114*  (GenBank accession number, NM_033197.2) | c.280A>T | p.Ile94Phe | Missense | 2.90 | Likely damaging | Innate immunity |
| *ADA*  (GenBank accession number, NM_000022.2) | c.22G>A | p.Asp8Asn | Missense | 4.00 | Likely benign | Immune defense |
