# Supplementary material for: Exome sequencing identifies NFS1 deficiency in a novel Fe-S cluster disease, infantile mitochondrial complex II/III deficiency
Source: Mol Genet Genomic Med. 2013 Nov 18;2(1):73–80. doi: 10.1002/mgg3.46 (PMC3907916; doi:10.1002/mgg3.46)
Supplement: Data S1 — Supplementary materials and methods. [file mgg30002-0073-sd3.docx]

Farhan et al., *Molecular Genetics & Genomic Medicine*

**Supporting Information for the article:**

**Exome sequencing identifies *NFS1* deficiency in a novel Fe-S cluster disease, infantile mitochondrial complex II/III deficiency**

**Supplementary materials and methods**

**Source of DNA**

Peripheral blood and biopsy samples were collected from the four children and the parents for autozygosity mapping using the Affymetrix^®^ Genome-Wide Human SNP Array 6.0. Genomic DNA from two affected individuals was evaluated with exome sequencing using the Illumina^®^ (San Diego, CA, USA) 2x100 base pair and the SureSelect Target Enrichment Kit (Agilent Technologies, Santa Clara, CA, USA). The genealogy of the affected family was constructed through interviews and community records.

**Enzymology assays**

Mitochondrial electron transport chain enzyme activities were measured using muscle and liver autopsy or biopsy samples taken from the first two affected individuals. Mitochondrial enzyme activities were measured using a previously described protocol with minor modifications [[Trounce et al., 1996](#_ENREF_12)].

**Histology**

Tissue from the muscle biopsy from affected individual IV-II was frozen and fixed in glutaraldehyde. Using standard methods, frozen sections were treated with the following stains and histochemical methods: Gomori modified trichrome (GMT); hematoxylin, phloxin and saffron (HPS); acid phosphatase; ATPase (at pH 4.2 and 9.6), cytochrome C oxidase (COX); reduced nicotinamide adenine dinucleotide-tetrazolium reductase (NADH); periodic acid-Schiff (PAS) and succinic dehydrogenase (SDH).  The tissues fixed in glutaraldehyde were post-fixed in osmium tetroxide and embedded in Epon; thin sections (silver interference) were mounted on standard grids and stained with uranyl acetate.

Routine pediatric autopsies were performed on individuals IV-I and IV-II. Representative areas from all organs were examined microscopically. The brains were retained and fixed in 20% formalin.  A standard neuropathological examination was carried out, including widespread sampling of various regions for microscopic examination. All sections were stained with hematoxylin and eosin; in addition, selected sections from the liver were stained with oil red O.

**Genotyping**

Genomic DNA extracted from three affected individuals, one unaffected sibling and both unaffected parents was genotyped for single nucleotide polymorphisms (SNPs) using Affymetrix^®^ (Santa Clara, CA, USA) protocols. 500 ng of genomic DNA was digested with two restriction enzymes, *Nsp*I and *Sty*I separately. All DNA fragments were ligated to sequence specific adaptors complementary to a PCR primer. Each digest was PCR amplified then combined and purified. Finally, PCR products were fragmented, labeled and hybridized to the Affymetrix^®^ Genome-Wide Human SNP Array 6.0. SNP genotypes were determined using the Birdseed v2 algorithm employed in the Affymetrix^®^ Genotyping Console^™^ Software 4.1.1 [[Korn et al., 2008](#_ENREF_5); [Rabbee and Speed, 2006](#_ENREF_10)].

**Autozygosity mapping**

GeneSpring GT v2.0 (Agilent Technologies, Santa Clara, CA, USA) software was used to identify regions of homozygosity that are identical by descent in family members. SNP allele frequencies from Caucasian controls were used to estimate logarithm of the odds (LOD) scores for each SNP. Location scores were calculated by summation of LOD scores of accumulated homozygous regions in the genome [[Broman and Weber, 1999](#_ENREF_2); [Lander and Botstein, 1987](#_ENREF_7)]. Homozygous regions unique to the affected individuals with a significantly high location score were treated as candidate loci.

**Whole-exome sequencing and mutation discovery**

Whole-exome sequencing was carried out using DNA from two affected individuals and performed on the Illumina Hiseq 2000 with 2x100 paired end chemistry according to protocols used by The Centre for Applied Genomics (TCAG) at the Hospital for Sick Children, Toronto, Ontario. The SureSelect Target Enrichment kit v1 (Agilent Technologies, Santa Clara, CA, USA) was used to enrich 40 Mbase of exome region. The Genome Analysis Toolkit [[DePristo et al., 2011](#_ENREF_3)] and CLC Bio Genomic Workbench were used to align the data to the human reference genome (Hg19) to produce a consensus sequence. Nonsynonymous variants within the autozygous region on chromosome 20p11.2-q13.1 and with a low minor allele frequency (MAF, <5%) according to NCBI dbSNP, 1000 Genomes or NHLBI ESP Exome Variant Server, were analyzed. We also surveyed the Human Gene Mutation Database (HGMD) for any reported mutations within the candidate genes [[Stenson et al., 2009](#_ENREF_11)]. Next, *in silico* analyses were carried out on two nonsynonymous variants identified by exome sequencing: p.Arg72Gln in *NFS1* (GenBank accession number, NM_021100.4.), and p.Arg167Cys in *CDH22* (GenBank accession number, NM_021248.2 ). To predict the state of pathogenicity of their respective mutations we used PMUT [[Ferrer-Costa et al., 2005](#_ENREF_4)], PolyPhen-2 [[Adzhubei et al., 2010](#_ENREF_1)], and SIFT [[Kumar et al., 2009](#_ENREF_6)]. ClustalW was used to determine conservation of amino acid residues across a set of diverged species by aligning species-specific homologs of both variants [[Larkin et al., 2007](#_ENREF_8)]. Eris software, which uses the Medusa modeling suite to predict the change in protein stability induced by mutations, was used to determine the stability of p.Arg72Gln in *NFS1* [[Yin et al., 2007](#_ENREF_13)]. We were unable to predict the protein stability associated with p.Arg167Cys in *CDH22* as a CDH22 crystalized protein structure with high homology to the human counterpart is currently unavailable. Clinical presentation of the affected individuals was suggestive of a mitochondrial disorder and thus, we used MitoCarta to determine whether the candidate genes were localized or targeted to the mitochondria (Supp. Fig. S1) [[Pagliarini et al., 2008](#_ENREF_9)].

To determine whether p.Arg72Gln in *NFS1* cosegregates with disease status in the pedigree, we performed confirmatory Sanger sequencing. Primers specific to amplify g.1573G>A in *NFS1* were 5’- TTG GTG GGT AGT TTT GTG GG -3’ and 5’- CCA TTT CTA CCT CCA TGC AC -3’. We also genotyped p.Arg167Cys in *CDH22*. Primers specific to amplify g.10682C>T in *CDH22* primers were 5’-CAA GTA CAC CAT CTC AGG CGA GG -3’ and 5’TCT TTT CTG TCC CTC CCC AGA GG-3’. PCR conditions consisted of initial denaturation at 95˚C for 5 minutes; 30 cycles of denaturation at 95˚C for 30 seconds; annealing at 58˚C for 30 seconds; extension 72˚C for 30 seconds and a final extension at 72˚C for 7 minutes. PCR products were loaded on a 1.5% agarose gel, purified and analyzed on an ABI 3730 DNA Sequencer (Applied Biosystems, Foster City, CA, USA).

**Population genotyping**

*NFS1* g.1573G>A, p.Arg72Gln was genotyped in 40 healthy Old Order Mennonite controls and 3,033 healthy individuals from an ethnically diverse cohort using a custom-designed TaqMan^®^ genotyping assay (Life Technologies, Carlsbad, CA, USA). Primers used by the custom probe assay were 5’- CGC TCA AGG AAA ATG ATC TGT TCT G -3’ and 5’- GGA GTG TGG GTT CCC ATA GTA GTT -3’. SNP genotyping was carried out using an allelic discrimination assay on the 7900HT Fast Real-Time PCR System (Life Technologies), and genotypes were identified using automated software (SDS 2.3; Life Technologies). Reactions were run in 5 µl volumes with an amplification protocol of 95˚C for 10 minutes; 50 cycles for 95˚C for 15 seconds and 60˚C for 1.5 minutes. For technical reasons, we genotyped g.10682C>T, p.Arg167Cys in *CDH22* in 40 Old Order Mennonite controls and 781 healthy individuals from an ethnically diverse cohort, using standard Sanger sequencing protocols as previously described, and restriction enzyme digestion using endonuclease *Aci*I to detect an abolished cut site, in accordance with the manufacturer’s instructions (New England BioLabs^®^, Ipswich, Massachusetts, USA.).

**Tissue culture and quantitative PCR**

Skin fibroblasts from patients and healthy controls were cultured in Petri plates with Dulbecco’s modified Eagle’s medium (GIBCO, Carlsbad, CA, USA) containing 10% fetal bovine serum until 60-70% confluency. RNA was isolated from fibroblast cells using the RNeasy Mini Kit according to the manufacturer’s instructions (Qiagen, Valencia, CA, USA). RNA quality was measured using the Agilent 2100 Bioanalyzer RNA 6000 Nano. RNA was reverse transcribed to cDNA using the High Capacity cDNA Reverse Transcription Kit (Life Technologies, Carlsbad, CA, USA) to carry out quantitative reverse-transcription PCR (qRT-PCR) using custom designed probes specific to the beginning and end of the *NFS1* transcript 5’ AGC GCA CTC TTC TAT CAG GTT TGG A 3’ and 5’ AGC TAC AAC TCC TCT GGA CCC CCG G 3’ and also for the *CDH22* transcript 5’ TGG ACC CCA AGA CCG GCG TAA TCC G 3’ and 5’ TTG ACA TCC AAG ACA ACA CCG CTG C 3’ (Life Technologies, Carlsbad, CA, USA). Gene expression assays were performed on the 7900HT Fast Real-Time PCR System (Life Technologies), and gene expression profiles were determined using automated software (SDS 2.3; Life Technologies).

**Protein quantification**

Protein expression was analyzed by western blotting. Proteins were isolated from cultured fibroblasts and resolved by 4-20% SDS-PAGE and transferred onto polyvinylidene difluoride membranes (Invitrogen). The membranes were blocked in TBS containing 0.1% Tween-20 and 5% fat-free dry milk for 1 hr and then incubated with anti-NFS1 (1:200, Santa Cruz Biotechnology, Santa Cruz, CA, USA) and anti-CDH22 (1:200, LifeSpan Biosciences, Inc, Seattle, WA, USA) overnight at 4˚C. The membranes were then incubated with peroxidase-conjugated anti-mouse secondary antibodies (1:10,000, Jackson ImmunoResearch Laboratories, Inc) for 1 hr, followed by NFS1 and CDH22 visualization with enhanced chemiluminescence-detection Luminol reagent according to the manufacturer's instructions (Santa Cruz Biotechnology).

**Co-immunoprecipitation**

Co-immunoprecipitations were performed in accordance with the manufacturer’s instructions with slight modifications (Pierce^®^ Co-Immunoprecipitation Kit, Thermo Scientific. Rockford, IL, USA). Co-immunoprecipitations were performed with anti-NFS1 (10 µg, Santa Cruz Biotechnology, Santa Cruz, CA, USA) followed by western analysis with anti-ISD11 (1:100, Aviva Systems Biology, San Diego, CA, USA).

**Supplementary References**

Adzhubei IA, Schmidt S, Peshkin L, Ramensky VE, Gerasimova A, Bork P, Kondrashov AS, Sunyaev SR. 2010. A method and server for predicting damaging missense mutations. Nat Methods 7(4):248-249.

Broman KW, Weber JL. 1999. Long homozygous chromosomal segments in reference families from the centre d'Etude du polymorphisme humain. Am J Hum Genet 65(6):1493-1500.

DePristo MA, Banks E, Poplin R, Garimella KV, Maguire JR, Hartl C, Philippakis AA, del Angel G, Rivas MA, Hanna M, McKenna A, Fennell TJ and others. 2011. A framework for variation discovery and genotyping using next-generation DNA sequencing data. Nat Genet 43(5):491-498.

Ferrer-Costa C, Gelpi JL, Zamakola L, Parraga I, de la Cruz X, Orozco M. 2005. PMUT: a web-based tool for the annotation of pathological mutations on proteins. Bioinformatics 21(14):3176-178.

Korn JM, Kuruvilla FG, McCarroll SA, Wysoker A, Nemesh J, Cawley S, Hubbell E, Veitch J, Collins PJ, Darvishi K, Lee C, Nizzari MM and others. 2008. Integrated genotype calling and association analysis of SNPs, common copy number polymorphisms and rare CNVs. Nat Genet 40(10):1253-1260.

Kumar P, Henikoff S, Ng PC. 2009. Predicting the effects of coding non-synonymous variants on protein function using the SIFT algorithm. Nat Protoc 4(7):1073-1081.

Lander ES, Botstein D. 1987. Homozygosity mapping: a way to map human recessive traits with the DNA of inbred children. Science 236(4808):1567-1570.

Larkin MA, Blackshields G, Brown NP, Chenna R, McGettigan PA, McWilliam H, Valentin F, Wallace IM, Wilm A, Lopez R, Thompson JD, Gibson TJ and others. 2007. Clustal W and Clustal X version 2.0. Bioinformatics 23(21):2947-2948.

Pagliarini DJ, Calvo SE, Chang B, Sheth SA, Vafai SB, Ong SE, Walford GA, Sugiana C, Boneh A, Chen WK, Hill DE, Vidal M and others. 2008. A mitochondrial protein compendium elucidates complex I disease biology. Cell 134(1):112-123.

Rabbee N, Speed TP. 2006. A genotype calling algorithm for affymetrix SNP arrays. Bioinformatics 22(1):7-12.

Stenson PD, Ball EV, Howells K, Phillips AD, Mort M, Cooper DN. 2009. The Human Gene Mutation Database: providing a comprehensive central mutation database for molecular diagnostics and personalized genomics. Hum Genomics 4(2):69-72.

Trounce IA, Kim YL, Jun AS, Wallace DC. 1996. Assessment of mitochondrial oxidative phosphorylation in patient muscle biopsies, lymphoblasts, and transmitochondrial cell lines. Methods Enzymol 264:484-509.

Yin S, Ding F, Dokholyan NV. 2007. Eris: an automated estimator of protein stability. Nat Methods 4(6):466-467.
